# Long-term outcome of surgery for lung cancer in Africa: a systematic review and meta-analysis

**DOI:** 10.3332/ecancer.2025.1951

**Published:** 2025-07-22

**Authors:** Adu Bukola Gift, Michael Joseph Otorkpa, Oluwatobi O. Olayode, Ebubechukwu David Joseph, Ademola Abdulhakeem, Efuetlateh John Paul Nchonganyi, Feziechi Chikelundu Anele, Akolade Akeem Habib, Fodop Samuel Ghislain Junior, Oluwanifemi O. Akintoye, Omoregbee Benjamin

**Affiliations:** Cardiothoracic Surgery, Surgery Interest Group of Africa, Lagos 100001, Nigeria

**Keywords:** Africa, lung cancer, lobectomy, surgery, lung cancer in Africa, cancer screening, oncology, meta-analysis, systematic review

## Abstract

**Background:**

Lung cancer is the leading cause of cancer death worldwide, with an estimated 1.8 million deaths in 2020. Despite the advancement of new treatment strategies that have emerged over time, surgery remains a very important aspect of cancer treatment. This study aims to highlight the long-term outcomes of surgery as well as the healthcare gaps in the diagnosis and treatment of Lung cancer in Africa by providing a comprehensive systematic review and meta-analysis.

**Methods:**

This systematic review was conducted using database searches from PubMed and Google Scholar to identify published data reporting on the surgical outcomes of lung cancer in Africa from inception till August 2024. We followed the Preferred Reporting Items for Systematic Reviews and Meta-Analysis guidelines to conduct this study. The primary outcomes of interest were overall mortality, 1- and 5-year survival rates, metastasis, morbidity and recurrence. Data were pooled together and analysed using a random-effect model for meta-analysis with R software. Out of a total of 381 articles identified, only eight papers met our inclusion criteria following deduplication and screening. The five countries with published research on our topic include Egypt, Kenya, Tunisia, Nigeria and Morocco, with a total sample size of 2150 patients.

**Results:**

The meta-analysis of the reported outcomes produced an overall mortality rate of 27%, a 1-year survival rate of 56%, a 5-year survival rate of 13%, metastases of 76.9%, morbidity of 7.7% and recurrence of 11.4%.

**Conclusion:**

The burden of lung cancer is relatively high across the African continent, with surgical treatment significantly underutilised due to several factors, including an inadequate number of skilled healthcare workers, limited cardiothoracic surgical services and the advanced stage at which most patients present. Nevertheless, there is room for improvement by addressing these gaps through targeted investments in cardiothoracic surgical training, research and infrastructure, alongside increased awareness of lung cancer and the benefits of screening services across Africa. These measures, combined with joint international and governmental funding efforts, could significantly improve survival outcomes.

## Introduction

Lung cancer remains one of the most devastating types of cancer globally, responsible for a substantial number of cancer-related deaths. According to the International Agency for Research on Cancer of the World Health Organisation, lung cancer is the most frequently diagnosed cancer worldwide, with 2.5 million new cases, representing 12.4% of all new cancer cases [1,2]. It is also the leading cause of cancer-related mortality, with 1.8 million people dying annually, accounting for 18.7% of all cancer-related deaths, more than the combined total cases of breast, colon and prostate cancer [1–3]. In Africa, the incidence of lung cancer varies significantly by region, with higher rates in South and North Africa compared to East, Middle and West Africa [3].

The burden of lung cancer in Africa is compounded by several factors, including low public awareness, a lack of screening programs for high-risk individuals, overburdened treatment centers and inadequate financial support [4–7]. Socioeconomic, geographic and healthcare infrastructure disparities present unique challenges to the effective management and treatment of lung cancer in the region. Additionally, late-stage diagnoses and limited access to healthcare resources result in poor outcomes for lung cancer patients [4–8].

Recent data and research highlight critical gaps in the surgical outcomes of lung cancer in Africa. Studies indicate that the continent faces unique challenges, such as a high prevalence of comorbidities, limited availability of diagnostic tools and a lack of trained healthcare professionals [4–6]. In the West African sub-region, for example, the majority of lung cancer patients present at advanced stages, with over 90% doing so at a point where curative intervention is no longer possible, as evident in the Ezemba *et al* [9] dataset, where merely 2% of patients were determined to be surgical candidates. Early detection and surgery are vital for ensuring a fair prognosis, but these are often unattainable due to the aforementioned barriers [7, 8, 10].

For resectable tumours, several factors significantly influence the survival outcomes of lung cancer patients. These include histological factors, such as tumour type, location, size, invasion and nodal status, as well as patient-related factors, such as age, gender, smoking status, performance status and initial treatment modality [11]. Despite advancements in predictive tools like nomograms, long-term surgical outcomes of lung cancer in Africa remain poorly understood due to a paucity of comprehensive data and systematic reviews [5, 11, 12].

This article aims to delve into the current state of surgical outcomes for lung cancer in Africa, drawing on recent data and research findings. By analysing these insights, we seek to understand the multifaceted barriers to effective surgical interventions and explore the effectiveness of treatments involving both surgical and non-surgical methods. The focus will be on examining mortality, morbidity and survival outcomes, particularly when surgery is involved, and how sociodemographic factors, treatment methods, combination therapy effects and healthcare system variables influence these outcomes. By providing a comprehensive analysis of long-term surgical outcomes, this study has the potential to inform clinical practice and policy-making in Africa, ultimately enhancing lung cancer management and improving patient outcomes. Understanding the unique factors influencing surgical success in this region is crucial for developing targeted interventions and optimising resource allocation.

## Methodology

We conducted a comprehensive search to identify relevant studies reporting on long-term surgical outcomes of lung cancer in Africa. This was done by the guidelines set out in the Preferred Reporting for Systematic Reviews and Meta-Analyses (PRISMA) across two databases: Google Scholar and PubMed.

We searched the PubMed database using structured Medical Subject Headings (MeSH): {Surgical Outcomes} and {Lung Cancer} and {Africa} and {Individual African Country} and we repeated the same for Google Scholar, ranging from inception to March 2024. Detailed information on the search strategy, including the keywords and MeSH terms utilised, is provided in the Table below:

### Inclusion criteria

Human studies conducted in any of the 54 African countries, involving either pediatric or adult patients who underwent surgery as a treatment for lung cancer and reported outcomes including mortality, were included in this review. Other inclusion criteria are original research papers, including controlled trials, case reports, prospective and retrospective studies and papers written in English.

**Table:** 

Database	Keywords	Hits
Google scholar	surgical outcome and lung cancer and Africa	100 (First 10 pages)
Google scholar	surgical outcome and lung cancer and (each of the 54 African countries).	5,400 (First 10 pages = 100 articles for each country searched)
Pubmed	Surgical outcome and lung cancer and Africa	54
Pubmed	Surgical outcome and lung cancer and each of the 54 African countries) Egypt = 92, Nigeria = 10, Seychelles = 0, Sao tome = 1, Cabo verde = 0, Comoros = 0, Djibouti = 0, Eswatini = 0, Mauritius = 0,Equatorial guinea = 0, Guinea bissau = 0, Lesotho = 0, Gabon = 0, Namibia = 1, Botswana = 4, Gambia = 2, Eritrea = 0, Mauritania = 0, Liberia = 0, Central african republic = 4, Libya = 4, Sierra leone = 1, Togo = 3, South sudan = 2, Tunisia = 0, Burundi = 0, Rwanda = 2, Guinea = 2, Zimbabwe = 0, Senegal = 3, Somalia = 1, Chad = 8, Zambia = 1, Malawi = 2, Burkina faso = 1, Mali = 5, Niger = 4, Cameroon = 2, Cote d'ivoire = 0, Madagascar = 2, Mozambique = 1, Ghana = 5, Angola = 0, Morocco = 26, Algeria = 3, Sudan = 4, Uganda = 5, Kenya = 3, South africa = 26, Tanzania = 2, Congo = 2, Ethiopia = 10	244

### Exclusion criteria

Exclusions included papers not in English, systematic reviews, commentaries, meta-analyses, abstracts, studies involving non-African populations and those with limited data. Additionally, studies not conducted on human subjects and those involving secondary lung cancer were excluded.

### Data extraction and analysis

Search results were exported to Rayyan.ai, a systematic review software, for deduplication and detailed screening. Initially, titles and abstracts were reviewed by nine independent authors, followed by a full-text screening of potentially relevant studies. To ensure consistency and accuracy, each paper was independently screened by at least two authors using the Newcastle–Ottawa scale. The qualities of the research papers evaluated include the selection process, comparability methods and the outcomes reported by each study. Articles that fulfilled the inclusion criteria were transferred to an Excel spreadsheet for data extraction.

### Variable of interest

The following information was extracted from the included papers for analysis and review: author, year of publication, journal, country, study design, follow-up period, age, sex, lung cancer stage at diagnosis, treatment received, type of surgery performed, additional supportive surgeries, chemotherapy administered, TNM staging, histological subtypes, metastasis, tobacco smoking, alcohol consumption and comorbidities (such as pneumonia, retroviral diseases, pleural effusion, hypertension, tuberculosis, Sickle cell disease, anemia, chronic obstructive pulmonary disease). Outcomes, including mortality, morbidity, the development of complications and recurrence, were also recorded. Appropriate summary statistics (mean, counts and proportions) were used to present the variables of interest, which were visually represented in tables.

## Results

### Distribution and types of studies

The initial search of electronic databases identified a total of 5,798 papers. After removing duplicates, 5,706 papers remained. Screening of titles and abstracts resulted in the exclusion of 5,653 papers, leaving 53 papers for full-text screening. An additional 45 papers were excluded during the full-text screening for not meeting the eligibility criteria. Ultimately, 8 papers met the predefined eligibility criteria and were included in this systematic review and meta-analysis.

**Figure 1.**
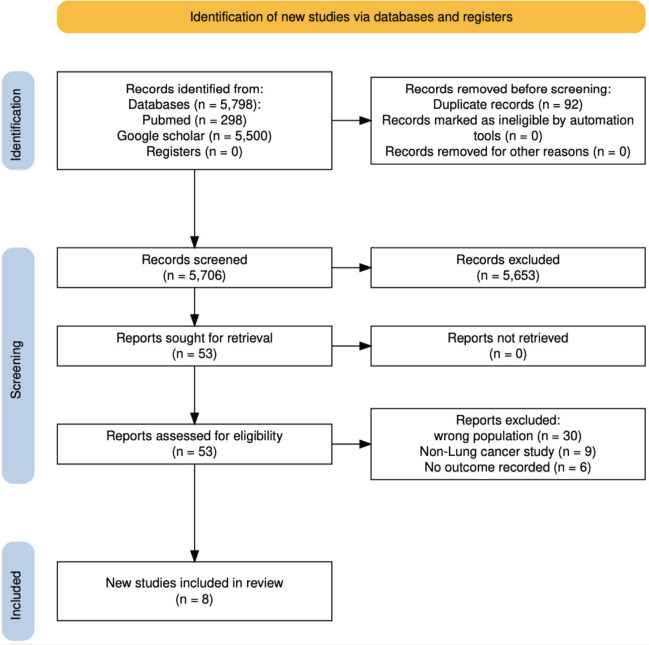
PRISMA flow diagram outlining the study selection process for inclusion in the systematic review and meta-analysis on surgical outcomes of lung cancer in Africa.

The 8 included papers were all retrospective studies. The studies originated from only five of the 54 African countries. Specifically, two (25%) of the studies were from Egypt, three (37.5%) were from Morocco and the remaining three studies were from Kenya (1), Nigeria (1) and Tunisia (Table 1) (1). The PRISMA flowchart showing the screening process is presented in Figure 1.

**Figure 2.**
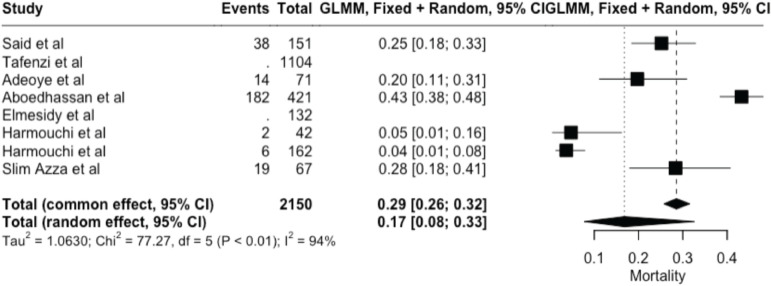
Forest plot showing pooled mortality prevalence (random effects model) – 17% (95% CI: 8–33%).

**Figure 3.**
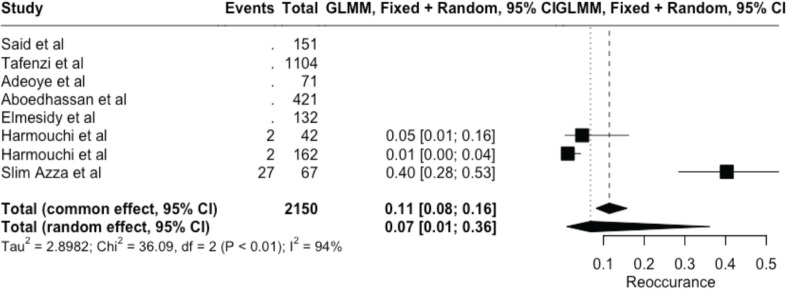
Forest plot showing pooled reoccurrence prevalence (random effect models) – 7% (95% CI: 1%–36%).

**Figure 4.**
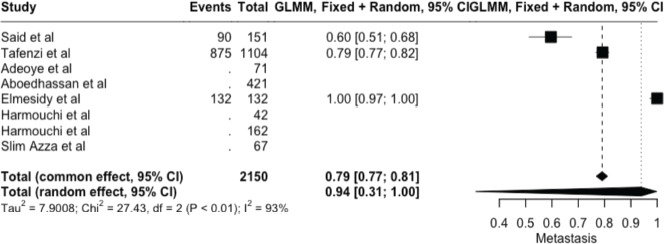
Forest plot showing the pooled metastasis prevalence (random effect models) – 94% (95% CI: 31%–100%).

The meta-analysis yielded key outcome indicators: a mortality rate of 17% (95% CI: 8%–33%), as shown in Figure 2. The recurrence rate following surgery was 7% (95% CI: 1%–36%) (Figure 3), while the prevalence of metastasis was notably high at 94% (95% CI: 31%–100%) (Figure 4). The wide confidence intervals observed in all three forest plots reflect substantial differences among the included studies, such as variations in patient populations, follow-up durations, and healthcare settings across different African countries. Even though the results vary widely, the findings are statistically significant (*p* < 0.05), This can help us understand the real challenges and outcomes of lung cancer surgery in Africa.

### Clinico-demographics

A total of 2,150 patients were included in the meta-analysis. A summary of the available demographic data is provided in Table 2. The average age, reported in six studies, was 58 years. Two studies indicated that, on average, 54.4% of their patients were under 60 years old, while 45.6% were over 60. The majority of the patients (82.3%) were male, with females comprising 17.7%. Regarding lifestyle factors and comorbidities, 59.7% had a history of smoking (either current or former smokers), 14.1% reported alcohol use, 1.3% had hypertension, 18% had retroviral disease, 1.8% had tuberculosis and 9% had chronic obstructive pulmonary disease (COPD).

Cancer staging was reported in several studies: four studies reported stage 1 cancer, with an overall prevalence of 1.97%, broken down into stage 1A (14.90%) and stage 1B (5.97%). Stage 2 cancer was reported in four studies, with an overall prevalence of 4.58%, including stage 2A (0.50%) and stage 2B (2.47%). Six studies reported stage 3 cancer (19.33%), with three of these differentiating between stage 3A (11.74%) and stage 3B (6.85%). Stage 4 cancer was reported in four studies, with a high prevalence of 81.50%; one of these studies further classified it into stage 4A (63%) and stage 4B (27%).

### Type of cancer and treatment received

The majority of cancers were adenocarcinomas, reported in seven studies, accounting for 48.82% of cases. Squamous cell carcinoma was also reported in seven studies, making up 12.90% of cases. Four studies reported large cell carcinoma at 5.93%. Additionally, two studies reported carcinoid tumours (4.90%) and undifferentiated cell types (4.94%). Other types of cancer included adenosis carcinoma (1%), neuroendocrine carcinoma (5.88%), adenosquamous carcinoma (0.81%), epidermoid carcinoma (22%) and a category labeled ‘others’ in four studies, accounting for 4.30% of cases (Table 3).

All studies reported surgical intervention, with 366 out of 2,150 patients (17%) undergoing surgery alone. Three studies reported that 28.10% of patients underwent surgery combined with other treatments. Only three papers specified the type of surgery performed, with the majority undergoing lobectomy. Four studies reported that 12.77% of patients received radiotherapy, while five studies indicated that 38.10% underwent chemotherapy (Table 4).

### Outcomes and complications

Mortality was reported in six studies, with an overall rate of 27%. Three studies reported a 1-year survival rate of 56%, and one study noted a 5-year survival rate of 13%. Metastasis was reported in two studies, with a rate of 76.9%, while morbidity was 7.7%. Recurrence was documented in three studies, with a rate of 11.4%.

Complication rates were also highlighted: bleeding was reported in two studies, occurring in 6.9% of cases, while wound infection occurred in 1%, and atelectasis in 3.9%. Additionally, one study reported a loss to follow-up rate of 74.7%, with other complications including disease progression (28.9%), postoperative hemothorax (0.9%), empyema (4.8%), prolonged air leak (3.1%) and persistent pleural pocket formation in 1.9% of cases (Table 5).

## Discussion

Our study represents the most up-to-date review of the surgical outcomes of lung cancer in Africa, highlighting the significant burden of the disease and identifying research gaps. We found that lung cancer affects men more than women across Africa, with geographic concentrations in five countries: Egypt, Morocco, Kenya, Nigeria and Tunisia. Egypt and Morocco account for 62.5% of the research output, indicating how underreported this disease is across Africa. Notably, the majority of studies included in this review originate from North Africa (Egypt, Morocco and Tunisia), with only two studies from Sub-Saharan Africa (Nigeria and Kenya). This geographic imbalance highlights the urgent need to promote more lung cancer research in Sub-Saharan Africa, where the burden of undiagnosed and untreated cancer cases also remains high. This imbalance underscores the disparities in resources and research capacity on the continent.

In addition to the geographic disparities, our review also sheds light on clinical characteristics and comorbidities affecting lung cancer patients in Africa. According to our findings, the mean age of lung cancer patients in Africa is 58 years, which is consistent with a Nigerian review [13] but significantly younger than the average age in high-income countries like the UK and USA, where it usually exceeds 70 years [14]. This discrepancy may be due to differences in healthcare access, demographics, risk factors and population structure. While younger patients generally have better surgical outcomes, the younger African population faces systemic barriers like delayed diagnosis, limited healthcare access and the use of alternative medicine such as herbs, which generally affects the outcome of lung cancer in affected patients [15].

Moreover, comorbidities represent a significant challenge in managing lung cancer in Africa. One of the included studies reported that 18% of patients had retroviral diseases and 9% had COPD [5], complicating management. This overlap has been reported to worsen prognosis and restrict treatment options, mirroring findings from the U.S, where lung cancer is the leading cause of cancer-related death amongst HIV-positive patients [16].

### Factors associated with late presentation

Another major finding in our study was the stage at which lung cancer is diagnosed, with 81.5% at stage IV, which, as we know, is a late stage where only a limited amount of treatment can improve the prognosis [4, 17]. This pattern of late diagnosis is also recorded in several other studies [11, 18, 19, 20]. The late stage of diagnosis stems from the fact that in Africa, there are limited screening programmes due to a shortage of specialised diagnostic tools as well as trained personnel [7, 21, 22], compounded by misdiagnoses such as tuberculosis, HIV and pericardial diseases [16, 23, 24, 25, 26]. In high-income countries, they have leveraged low-dose CT scans to detect lung cancer earlier [27, 28], which research has shown improves survival rates by early diagnosis, leading to better surgical outcomes [29, 30]. It has also been noted that the limited availability of tissue samples for lung cancer confirmation complicates the high prevalence of late-stage diagnoses in Africa. The low rates of confirmed cases from biopsies demonstrate this [31]. Additionally, the lack of early detection in Africa is further worsened by the limited awareness of lung cancer symptoms due to its non-specific presentation, risk factors among patients and attitudes towards lung cancer [27, 32, 33, 34].

### Challenges of lung cancer surgery in Africa

Lung cancer management faces both financial and surgical capacity limitations. Due to the limited information in our study, funding was not directly addressed, but it is well known that many African lung cancer patients are unable to pay for treatment at the time of diagnosis due to high costs and Africa's unstable economy. Healthcare investments must continue to expand, which requires fiscal control and economic expansion to support healthcare advancements. African governments must make smart healthcare policy decisions to increase CTS infrastructure, training and retention. There is also a need to seek out and allow alternate finance sources to thrive to resolve CTS access inequalities, which will require coordinated measures and coordination between authorities, medical facilities and foreign associates [35].

Advanced surgery, especially minimally invasive operations, requires specialised training and institutional resources. Limited surgical treatments show the need for expanded thoracic surgery training programmes to improve lung cancer care in Africa. In addition to poor training, Africa lacks cardiothoracic surgeons to satisfy demand and CTS education is compromised. Optimal results will come from expanding cardiothoracic surgical capacity to be complemented by rising diagnostics, anaesthesia, perioperative care, rehabilitation and palliative services. The Lancet Oncology Commission (2023) stated, ‘Comprehensive systems are needed for cancer surgical care to improve outcomes [36].’

As noted by our study, surgery was performed in only 17% of cases, with lobectomy being the most common procedure. The low surgical intervention rate reflects the advanced disease stages at diagnosis. The limited availability of minimally invasive surgeries in Africa further restricts treatment options, leading to reliance on other treatment choices such as chemotherapy (38.10%) and radiotherapy (12.77%), although their efficacy is also compromised by the late presentation of the disease.

### Common histological types in Africa and how they influence surgical outcomes

The majority of cancers in this study were adenocarcinomas (48.82%), followed by squamous cell carcinoma (12.90%), which reflects similar findings in different African studies [5, 11, 18, 19, 37, 38, 39] and also in studies conducted in Spain and the USA [40, 41]. It is also documented that adenocarcinomas are the most common subtype of lung cancer among non-smokers, with SCLC strongly linked with smoking habits. Thus, it could be postulated that due to a global drop in smoking patterns, the prevalence of SCLC in smokers has been decreasing. Although studies have indicated that squamous cell carcinoma and adenocarcinoma of the lungs tend to have better survival rates [36], the trend of late presentation has significantly affected the treatment and outcome of lung cancer in Africa.

### Treatment options for lung cancer in Africa

Treatment options for lung cancer include chemotherapy, radiotherapy, targeted therapy and surgery. In Africa, the most commonly offered treatments are chemotherapy and combination therapies (chemotherapy plus radiotherapy) [5, 11, 18, 19, 39]. However, surgical resection remains the most effective treatment for lung cancer, especially in cases diagnosed at an early stage (localised tumours), though only a limited number of cases meet this criterion [42, 43]. This is primarily because most lung cancer cases in Africa and other regions present at advanced stages (stage 3b and stage 4), where surgery offers little to no benefit. Harmouchi *et al* [43] in their study conducted in Morocco, reported that all lung cancer cases undergoing surgery involved lobectomy and mediastinal lymphadenectomy, with fewer than 10% undergoing parietectomy of the chest wall. This was feasible because these cases did not present at advanced stages [44].

### Outcomes (Mortality, recurrence, complications and follow-up)

The reported mortality rate of 27%, 5-year survival rate of 13%, high recurrence rate (11.4%) and high complication rates (76.9% metastasis) in our study are alarmingly low. All of these underscore the challenges in managing lung cancer in Africa. Additionally, the high rate of loss to follow-up (74.7%) poses significant challenges to continuous care, which is crucial for improving survival outcomes. One such study, conducted in 2019 by Harmouchi *et al* [43] in Morocco with an 8-year follow-up period, observed a mortality rate of less than 10% following surgery, a recurrence rate of under 5% and approximately 15% morbidity [44]. These findings suggest that early detection of lung cancer, when surgical intervention is still possible, significantly improves survival rates. A study conducted in Sweden by Myrdal *et al* [44] also concluded that morbidity and mortality rates for lung cancer decrease following surgical interventions in patients presenting at an early stage of the disease [45].

### Recommendation to improve poor outcomes of lung cancer in Africa

Organisations such as the WHO and African societies like the West African College of Surgeons (WACSs) and the College of Surgeons of East, Central and Southern Africa (COSECSA) in collaboration with governments have a great role to play in strengthening the management and improving the outcome of lung cancer in Africa by expanding cardiothoracic surgery training programs, providing continuing medical education and fostering regional collaboration, these societies can help address the critical shortage of skilled thoracic surgeons. Furthermore, their involvement in setting regional surgical standards, accrediting training centers and advocating for cancer-focused surgical policy can facilitate the establishment of sustainable thoracic oncology services. Through partnerships with academic institutions, global surgical networks and local governments, WACS and COSECSA can also lead efforts in research, data collection and guideline development, stepping up efforts in raising awareness about the signs and symptoms of lung cancer, encouraging continent-wide affordable or free screening tests and discourage the use of tobacco products all of which are essential to improving lung cancer outcomes in Africa. It has been established that low-dose CT scans have been shown to increase surgical volumes and improve patient outcomes by enabling earlier detection of lung cancer [29, 30]. To enhance screening uptake, it is essential to understand the factors that motivate individuals to participate in such programmes [45]. Integrating smoking cessation interventions into screening can further reduce lung cancer risk, particularly as nearly 60% of patients in our study were smokers [47]. Additionally, educating primary care providers on screening guidelines and the effectiveness of available tests is vital for encouraging patient participation [48]. In line with this, lung cancer screening recommendations in Southern Africa underscore the importance of early detection in improving patient outcomes [49]. While encouraging a robust lung cancer screening program, we must also critically assess its feasibility within the African context. Challenges such as limited access to diagnostic tools, surgeons, post-operative care, radiotherapy and anti-cancer medications complicate the implementation of low-dose CT screening [50]. Though screening may help with early detection, it is still crucial to assess if the healthcare systems are sufficiently prepared to handle the rising need for services after a lung cancer diagnosis. Therefore, improving healthcare infrastructure is essential to assist screening initiatives as well as subsequent care pathways for patients diagnosed with lung cancer [17].

Addressing the shortage of thoracic surgeons in Africa requires investment in developing local surgical training programs and enhancing surgical skills through collaboration and targeted training opportunities abroad. Noteworthy is the need for universal health coverage and support for the most vulnerable in society. Funding from government agencies, NGOs and external partners will go a long way in achieving this aim, as it was established that uninsured patients and patients treated in lower-standard hospitals, as well as other socio-economic factors, contribute to poorer outcomes [51].

Finally, setting up more cancer care facilities across the continent to encourage easy access to specialised care can help reduce the burden of late diagnoses and improve treatment outcomes for patients.

## Conclusion

Several factors affect the long-term surgical outcomes of lung cancer in Africa, including late-stage diagnosis, limited access to advanced surgical techniques and the lack of specialised cardiothoracic surgeons. This study has demonstrated that the burden of lung cancer is significantly higher among younger patients as compared to older patients in high-income countries, with additional factors like delayed diagnosis and comorbidities, which further deteriorate treatment outcomes. The low rate of surgical interventions, particularly minimally invasive surgeries, reflects the advanced stages at which most patients are diagnosed and the lack of skilled surgeons to perform them, which ultimately leads to poor survival rates and high mortality.

To improve these outcomes, it is important to address the gaps in early detection through widespread screening programmes throughout Africa, enhance surgical training and improve access to essential surgical equipment. Additionally, targeted funding and international collaboration are needed to build the necessary infrastructure and human resource capacity to manage lung cancer effectively. By implementing these solutions, we can hope to improve the long-term surgical outcomes for lung cancer patients in Africa, ultimately reducing the disease's mortality and improving quality of life.

## Conflicts of interest

The authors declared no conflicts of interest.

## Funding

There was no external funding available for this study.

## Figures and Tables

**Table 1.** Characteristics of the papers included in the review.

Authors	Country	Year of publication	Study design	Sample size	Journal
Said, Nur Swaleh; Degu, Amsalu;	Kenya	2023	Retrospective study	151	*Cancer Medicine*
Tafenzi, Hassan Abdelilah; Choulli,	Morocco	2023	Retrospective study	1,104	*BMC Cancer*
Adeoye, Peter; Desalu, Olufemi;	Nigeria	2021	Retrospective study	71	*West African Journal of Medicine*
Aboelhassan, Rasha; Sobeih, Mohamed Emam	Egypt	2023	Retrospective study	421	*Sage Journals*
Elmesidy, Salah; Zawam, Hussam; Hassan,	Egypt	2016	Retrospective study	132	*Research in Oncology*
Harmouchi H, Lakranbi M, Issoufou I, Belliraj L	Morocco	2019	Retrospective study	42	*Clinics In Surgery*
Harmouchi, H; Ammor, FZ; Bellirej,	Morocco	2020	Retrospective study	162	*Open Journal of Surgery*
Slim, Azza; Kamoun, Hela; Hadidene, Yasmin;	Tunisia	2021	Retrospective study	67	*Journal of the Tunisian Society of Medical Sciences*

**Table 2.** Clinicodemographics.

Characteristics	Number of papers	Total population	% or Mean
Demographics			
Age			
Absolute mean	6	1,867	58
<60 years	2	154/283	54.4%
≥60 years	2	129/283	45.6%
Gender			
Male	8	1,770/2,150	82.3%
Female	8	380/2,150	17.7%
Comorbidities			
Smoking	8	1,283/2,150	59.7%
Alcohol	2	177/1,255	14.1%
Hypertension	1	2/151	1.3%
Retroviral diseases	1	7/151	18%
Pneumonia	1	4/151	10.3%
Pleural effusion	1	4/151	10.3%
Sickle cell disease	1	1/151	2.6%
Tuberculosis	2	4/218	1.8%
Anemia	2	28/1,255	2.2%
COPD	1	6/67	9%

**Table 3.** Cancer characteristics.

Stage 1	4	27/1,374	1.97%
Stage 2	4	63/1,374	4.58%
Stage 3	6	347/1,795	19.33%
Stage 4	4	1121/1,374	81.50%
Stage 1A	1	10/67	14.90%
Stage 1B	1	4/67	5.97%
Stage 2A	2	6/1,171	0.50%
Stage 2b	2	29/1,171	2.47%
Stage 3A	3	187/1,592	11.74%
Stage 3B	3	109/1,592	6.85%
Stage 4A	1	699/1,104	63%
Stage 4B	1	300/1,104	27%
Histology			
Nonsmall cell	4	623/776	80.20%
Small cell	0	0	0
Squamous cell	7	258/1,999	12.90%
Adenocarcinoma	7	976/1,999	48.82%
Large cell carcinoma	4	41/691	5.93%
Carcinoid	2	10/204	4.90%
Undifferentiated	2	14/283	4.94%
Others	4	23/534	4.30%
Adenosis carcinoma	1	1/67	1%
Neuroendocrine carcinoma	1	65/1,104	5.88%
Adenosquamous cell carcinoma	1	9/1,104	0.81%
Epidermoid carcinoma	1	243/1,104	22.00%

**Table 4.** Treatment pattern.

Treatment patterns			
Surgery only	8	366/2,150	17%
Chemotherapy	5	876/1,879	38.10%
Surgery + other treatment	3	180/639	28.10%
Radiotherapy	4	231/1,808	12.77%

**Table 5.** Complications and outcomes.

Complication			
Bleeding	2	14/204	6.9%
Empyema	1	2/42	4.8%
Wall infection	2	2/204	1%
Atelectasis	2	8/204	3.9%
Prolonged air leak	1	5/162	3.1%
Persistent pleural pocket	1	3/162	1.9%
Postoperative haemothorax	1	1/162	0.6%
Other outcome			
Lost to follow up	1	53/71	74.7%
Progression	1	122/421	28.9%
**Outcomes**			
Mortality	6	247/914	27%
Survival (1 year)	3	164/289	56.7%
Survival (5 year)	1	9/67	13%
Metastasis	2	965/1,255	76.9%
Morbidity	2	39/504	7.7
Recurrence	3	31/271	11.4%
